# Acupuncture Prescription Based on Thermal-Sensitized Acupoints for the Treatment of Knee Osteoarthritis: Protocol for a Randomized Controlled Pilot Trial

**DOI:** 10.2196/81837

**Published:** 2026-01-28

**Authors:** Xue-Zhou Wang, Tao-Tao Lv, Kua-Yue Zhang, Zhuo-Ya Chen, Li-Na Qin, Wei-Juan Gang, Wei-Heng Chen, Bao-Hong Mi

**Affiliations:** 1 The Third Affiliated Hospital of Beijing University of Chinese Medicine Beijing China; 2 Institute of Acupuncture and Moxibustion, China Academy of Chinese Medical Sciences Baijing China; 3 Engineering Research Center of Chinese Orthopaedics and Sports Rehabilitation Artificial Intelligent, Ministry of Education Beijing China

**Keywords:** knee osteoarthritis, acupuncture, infrared thermography, acupoint, pilot

## Abstract

**Background:**

Treatment options for knee osteoarthritis (KOA) remain limited. Although previous studies suggest that acupuncture can alleviate pain and functional impairment associated with KOA, there is significant variation in acupoint selection across studies and a lack of objective criteria to guide this choice. Infrared thermography (IRT) is recognized as a reliable method for identifying inflammatory regions in KOA. Our previous research revealed abnormal skin temperature at specific acupoints and surrounding areas in the lower limbs of patients with KOA, indicating potential treatment targets. However, there is still insufficient evidence to support their clinical application.

**Objective:**

This pilot trial aims to assess the feasibility of conducting large-scale studies in the future and to provide preliminary evidence of the effects of acupuncture applied at IRT-guided thermal-sensitized acupoints (TAs) for the treatment of KOA.

**Methods:**

This will be a randomized, controlled pilot trial with participant blinding. A total of 60 patients with KOA will be randomly allocated in a 1:1 ratio to either the TA group or the conventional acupoint group. Both groups will receive 3 acupuncture sessions per week for 4 weeks. In the TA group, acupoint selection will be based on points with abnormal skin temperature identified by IRT using real-time surface projection technology. In the conventional acupoint group, acupoint selection will follow the established prescription validated in previous studies. The primary outcome will be feasibility, assessed using a traffic light system to evaluate recruitment, retention, intervention completion, and completion of other outcome measures. Secondary outcomes will include the visual analog scale for pain, the Western Ontario and McMaster Universities Osteoarthritis Index function subscale, the credibility and expectancy questionnaire, skin temperature, and safety.

**Results:**

The study has been approved by the ethics committee of the Third Affiliated Hospital of Beijing University of Chinese Medicine (BZYSY-2024KYKTPJ-44) and registered in the International Traditional Medicine Clinical Trial Registry. The first participant was enrolled on February 25, 2025, and a total of 30 participants had been enrolled by January 1, 2026. All enrollment and follow-up are expected to be completed by June 2026.

**Conclusions:**

The findings of this study will provide evidence on the feasibility and preliminary clinical effects to support the future adoption of acupuncture prescriptions based on TAs for the treatment of KOA.

**Trial Registration:**

International Traditional Medicine Clinical Trial Registry ITMCTR2025000019; https://itmctr.ccebtcm.org.cn/mgt/project/view/-5870917416012215224

**International Registered Report Identifier (IRRID):**

DERR1-10.2196/81837

## Introduction

Knee osteoarthritis (KOA) is the most prevalent form of osteoarthritis, primarily characterized by chronic pain and functional impairment [1]. Current treatment options remain limited. Although pharmacological therapies may provide short-term symptom relief, their long-term use is often constrained by potential cardiovascular and gastrointestinal risks [2]. Acupuncture is a widely used nonpharmacological intervention and has been proven to be superior to sham acupuncture in certain studies [3,4]. However, these benefits are not consistently observed, particularly in studies conducted outside China [5,6]. Consequently, the American College of Rheumatology and the American Academy of Orthopaedic Surgeons [7,8] have generally assigned acupuncture a limited strength of recommendation in clinical practice guidelines.

The effects of acupuncture can be attributed to both acupoint stimulation and patient expectations [9]. Acupoints are central to acupuncture treatment; however, there is considerable variation in acupoint selection across studies on KOA. Guided by traditional acupuncture theory, more than 40 acupoints have been considered for the treatment of KOA [10,11]. The lack of clear criteria for acupoint selection has raised concerns regarding the adequacy of acupuncture protocols, as most studies fail to provide objective justification for their choice of acupoints [12]. The importance of the many available acupoints in traditional acupuncture theory remains unclear, as meta-analyses have shown no correlation between the number of acupoints used and clinical effect [9]. Experimental studies further support this observation, as animal studies using only 1 to 2 fixed acupoints often conclude that acupuncture is effective [13,14]. One possible explanation is that the effects of acupuncture may not depend on specific acupoints per se but rather on broader subcutaneous tissue stimulation.

Inflammatory diseases are closely related to thermoregulation. The severity of KOA has been shown to correlate with the temperature of specific areas surrounding the knee joint [15,16]. When abnormal temperature changes occur at or near acupoints, it is referred to as acupoint sensitization. These abnormal skin temperature variations may be associated with autonomic nervous system function and metabolic activity, with certain acupoints exhibiting more pronounced temperature changes than nonacupoint regions [17]. Recent anatomical studies [18] have demonstrated a notable overlap between acupoints and perforating cutaneous blood vessels. Using infrared thermography (IRT), it is possible to detect temperature changes in these blood vessels beneath the acupoints [19]. Our previous research [20] indicates that, compared with individuals without KOA, patients with KOA exhibit thermal sensitization at certain acupoints of the lower limbs, showing characteristics of localized and regional thermal consistencies. In the treatment of peripheral facial paralysis, acupuncture intervention at these temperature-abnormal points is effective [21]. However, in the case of KOA, there is currently limited evidence supporting the effectiveness of this acupuncture approach.

Compared with conventional acupoint (CA) selection, thermal-sensitized acupoints (TAs) may offer improved therapeutic effects. Under IRT guidance, these acupoints can be flexibly selected according to individual differences, while the procedure remains easy to standardize. IRT is convenient and noninvasive, which may result in high acceptability without imposing additional burden on participants. Therefore, this study aims to conduct a pilot trial comparing acupuncture using IRT projection technology for acupoint selection with CA therapy for KOA. The goal is to explore the feasibility of conducting a large-scale clinical trial and to assess differences in effectiveness between the two acupoint selection methods in the treatment of KOA.

## Methods

### Study Design

This will be a single-center randomized controlled pilot trial conducted at the Third Affiliated Hospital of Beijing University of Chinese Medicine. Participants will be randomly assigned to the TA group and the CA group in a 1:1 ratio. This study will be reported according to the Standard Protocol Items: Recommendations for Interventional Trials (SPIRIT) guidelines [22] (Multimedia Appendix 1).

### Participants

Before any study-specific procedures are initiated, individuals expressing interest in participation will receive a comprehensive explanation of the study aims, procedures, and relevance. Information regarding potential benefits, risks, and discomforts will be communicated clearly to support informed decision-making. Participation is voluntary, and participants may withdraw at any time. Individuals who decline participation will be informed of available conventional treatment options upon request. Written informed consent will be obtained before eligibility screening.

As this study compared acupuncture applied at TAs with that applied at CAs, the inclusion and exclusion criteria were based on those used in our previous studies evaluating the effects of conventional acupuncture [23]. The inclusion criteria are as follows: (1) age 45 to 75 years, (2) meeting the American College of Rheumatology diagnostic criteria for KOA [24], (3) reporting knee pain for more than 3 months, (4) having undergone radiological examination within the past 6 months indicating a Kellgren-Lawrence grade II or III [25], (5) having a score ≥40 mm in the visual analog scale (VAS) [26] during the past week, and (6) being willing to sign the informed consent.

Patients will be excluded if they meet any of the following conditions: (1) a history of knee joint surgery or plans for knee joint surgery (eg, knee replacement or knee arthroscopy); (2) knee pain attributed to other pathologies (eg, meniscus tear, rheumatoid arthritis, joint cavity infection, malignancy, gout, or lumbosacral disease with symptom of lower extremity); (3) a history of arthroscopy within the past 1 year or intra-articular injection within the past 6 months in the evaluating knee; (4) receipt of acupuncture in either knee within the past 6 months; (5) severe acute or chronic organic diseases or neuropsychiatric disorders; (6) disorders of coagulopathy (eg, hemophilia); (7) presence of a pacemaker or epilepsy; (8) pregnancy, planned pregnancy, or lactation; and (9) participation in other clinical studies within the past 1 month.

### Randomization and Blinding

The randomization sequence will be generated by an independent statistician using Stata (version 17.0; StataCorp). To conceal the contents, opaque envelopes will be used for distribution. These envelopes will be stored and managed by independent researchers not involved in other aspects of the trial. After including participants who met the criteria and obtaining their informed consent forms, random envelopes will be opened sequentially. The acupuncturists will be aware of group assignments, whereas participants and outcome evaluators will remain blinded.

### Interventions

#### Overview

The difference between the two intervention groups will be limited to the selection of acupoints only. All participants will undergo 4 weeks of acupuncture intervention accompanied by electrical stimulation. The treatment will be administered 3 times per week, with each session lasting 30 minutes. Ideally, sessions will be scheduled every other day, and administration of 2 sessions within a 24-hour period will be prohibited. Over a period of 4 weeks, a total of 12 treatments will be provided. Participants will assume a supine position on the treatment bed, and sterile, single-use needles will be used.

After the needle is inserted, the acupuncturists will perform lifting-insertion and twisting-rotating operations for at least 10 seconds to achieve the *deqi* sensation. The electroacupuncture instrument will then be connected, and a sparse-dense wave will be applied. Current intensity will be adjusted according to the participant’s comfort level. The intervention will be delivered by 3 acupuncturists with more than 3 years of clinical experience.

During the trial, participants will be discouraged from undergoing any other treatments that might affect their symptoms, including medications (such as nonsteroidal anti-inflammatory drugs, opioids, etc) and other physical therapies. Participants may use diclofenac sodium enteric–coated tablets (Voltaren, Beijing Novartis Pharma Co., Ltd) for severe pain that cannot be relieved, except within 48 hours before any assessment. If rescue medication is required, participants will receive up to 6 tablets, take 1 tablet 3 times daily, and discontinue use once symptoms improve, returning any remaining tablets to the study staff. Any treatment other than the trial intervention will be recorded, including timing and dose.

#### TA Group

**Figure 1:**
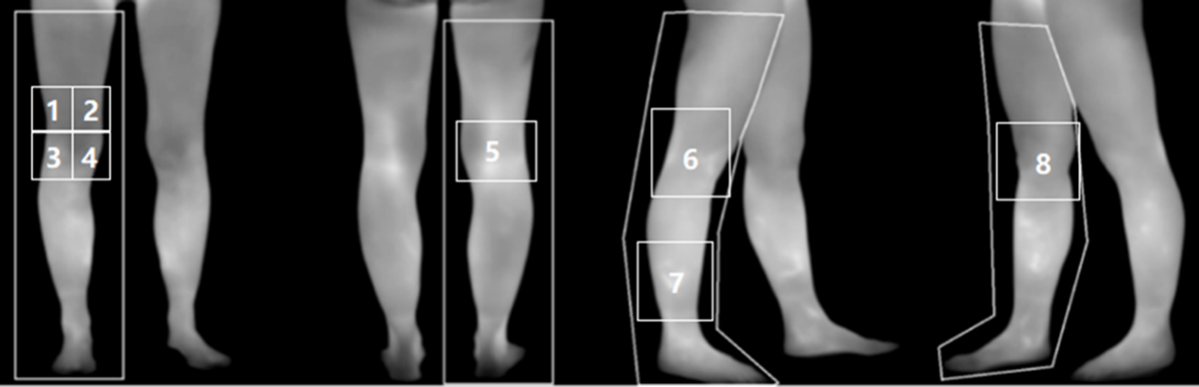
The 8 regions on the lower limbs used for acupoint selection.

Acupoint selection in the TA group is based on the points with the highest skin temperature in 8 specific lower limb regions (Figure 1). Our previous studies have indicated that, in patients with KOA, regional temperature increases occur in the lower limb acupoint areas as shown in Figure 1. These increases present a diffuse pattern resembling a “surface spread,” suggesting the presence of inflammatory reactions near the acupoint regions [20]. The areas with the highest temperatures are likely to correspond to locations of the most intense inflammatory responses. Therefore, these temperature-abnormal points will be selected as intervention acupoints in the TA group.

**Figure 2:**
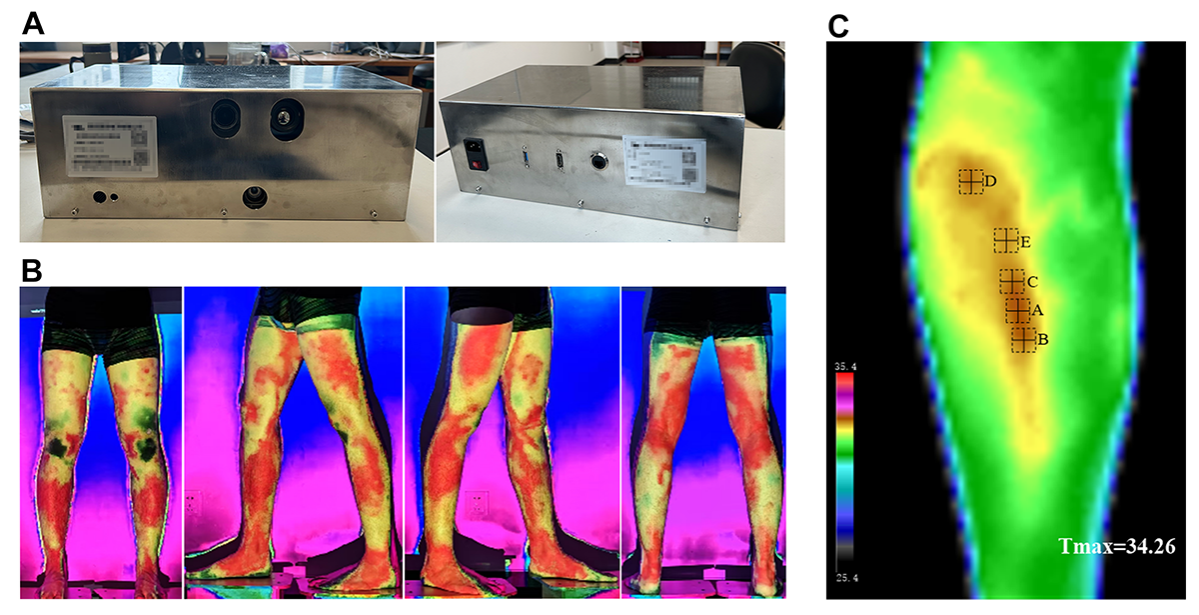
Infrared thermographic projection system for acupoint selection. (A) Infrared thermographic projection device; (B) real-time infrared image projected onto the body surface; (C) skin temperature recognition system. The squares indicate the automatically identified points with the highest skin temperature within the region.

Acupoint selection will be performed using a system established by the research team, consisting of a custom-designed IRT image projection device (patent authorization number CN214748459U) and an auxiliary acupoint selection software system (patent authorization number CN114582489B). This system can project infrared images onto the body surface in real time (Figures 2A and 2B), monitor temperature extremes within the region, and visually present the data (Figure 2C).

The parameters of the IRT device are as follows: (1) a resolution of 640×512, (2) a temperature sensitivity of 0.05 ℃, (3) a field of view of 33.5°×26.9°, and (4) manufactured by Airui Optoelectronics Technology Co Ltd. The projector parameters are as follows: (1) a luminance of 1400 ANSI lumens, (2) a lens resolution of 1920×1080 (4K resolution), (3) the HK-610 model, and (4) an IRT image projection distance of 1 to 2 meters.

The background filtering method adopted is a bimodal filter. This method iteratively and statistically analyzes the 2D data of the image, calculates the distribution frequency curve for any temperature value, and applies a Gaussian smoothing filter. The image filtering threshold is automatically determined by locating the 2 peaks of the distribution curve, thereby filtering the raw image data and retaining only the target region of interest for the study. The maximum regional temperature value is calculated using an improved maximum value method.

Participants in the TA group will undergo IRT inspection before the first intervention session to determine the initial intervention points. Thereafter, IRT inspection will be repeated before the first session of each subsequent treatment week to update the acupoints accordingly. Therapists will record the treatment sites using photographs to ensure consistency within each treatment cycle.

#### CA Group

The CA group acupoint selection adopts a previously validated acupuncture intervention protocol [3]. It includes 5 fixed acupoints as follows: Dubi (ST35), Neixiyan (EX-LE5), Ququan (LR8), Xiyangguan (GB33), and an Ashi point (the point at which the patient experiences the most pain). Additionally, 3 optional acupoints will be selected based on the acupuncturist’s diagnosis from a pool of 22 possible acupoints. Detailed acupoint selection information is provided in Table S1 in Multimedia Appendix 2.

Participants in the CA group will undergo IRT inspection before the first intervention session each week, consistent with the procedure used in the TA group. However, these assessments will be intended solely for blinding purposes and will not inform the selection of acupuncture points.

### Outcomes

#### Primary Outcome

The primary outcome will be feasibility. Feasibility will be assessed throughout the trial period, including recruitment rate, retention rate, intervention adherence, and outcome assessment. A traffic light system will be used to evaluate feasibility (Table 1). Outcomes exceeding the upper threshold will be considered feasible; those falling between the upper and lower thresholds will be interpreted as having limited feasibility, and protocol modifications for each outcome will be considered in future studies to enhance feasibility to an acceptable level. If any outcome falls below the lower threshold, the trial will be considered infeasible. In such cases, the results will primarily be used to analyze the reasons for trial failure.

**Table 1:** Feasibility assessment using a traffic light system.

Dimension	Lower threshold (%)	Upper threshold (%)
Recruitment rate	30	60
Retention rate^a^	70	80
Intervention completion rate^b^	70	90
**Outcome completion rate**		
VAS^c^ score	60	70
WOMAC^d^ function	60	70
Credibility and expectancy questionnaire	60	70
Skin temperature	60	70

^a^Including follow-up.

^b^Defined as completing at least 80% (10 sessions) of the intervention with no major protocol deviations.

^c^VAS: visual analog scale.

^d^WOMAC: Western Ontario and McMaster Universities Osteoarthritis Index.

#### Secondary Outcomes

##### Pain

Knee pain will be assessed using the VAS, a 100-mm scale in which one end represents “no pain” and the other represents “the worst pain imaginable” [26]. The VAS will be assessed at baseline and at weeks 2, 4, 8, and 12.

##### Function

Functional impairment will be evaluated using the Western Ontario and McMaster Universities Osteoarthritis Index (WOMAC) function subscale [27], which consists of 17 items rated on a 0 to 4 scale, with total scores ranging from 0 to 68. Higher scores indicate worse knee function. Assessments will be conducted at baseline and at weeks 2, 4, 8, and 12.

##### Expectancy

Expectancy will be measured using the credibility and expectancy questionnaire [28], which consists of 6 items. The first 3 items assess the credibility of the intervention, and the remaining 3 assess participants’ expectancy regarding the subjective effect. The questionnaire will be administered within 5 minutes after the first treatment session. Standardized scoring will be applied by adjusting each item score to have a mean of 0 and an SD of 1. The credibility score will be calculated as the sum of the standardized scores of the first 3 items, and the expectancy score, as the sum of the last 3 items.

##### Skin Temperature

Skin temperature at acupoints on the lower limb region will be measured using a portable medical IRT device. The measurement method will be the same as that used in our previous study [29] (Multimedia Appendix 2).

##### Safety

The safety of both interventions will be evaluated based on adverse events. Any adverse events will be documented within 24 hours of occurrence, and their relationship to the intervention will be determined by the research team. When necessary, the researcher responsible for maintaining the randomization sequence will perform unblinding. Serious adverse events will be referred to the principal investigator for further evaluation and management. Common adverse events related to acupuncture include pain, local hematoma, infection, and dizziness.

Feasibility, skin temperature, and safety outcomes will be assessed and documented by investigators. For participant-reported outcomes, investigators will first provide standardized explanations of questionnaire items, after which participants will complete the questionnaires independently, with minimal interaction to reduce potential influence. During the follow-up period, the questionnaire assessments will be conducted by telephone to improve retention.

### Sample Size

As this is a pilot study without previous evidence to estimate the difference between the current two groups, we selected a sample size of 30 participants per group based on the CI upper limit method [30]. Simulation studies suggest that this sample size provides stable estimates of variability, resulting in an 80% to 90% likelihood that a subsequent definitive trial will reach adequate power [31]. Assessment of treatment effects will be exploratory and intended to inform a future confirmatory trial, with CI bounds used because point estimates have limited precision in small samples [32]. In total, 60 patients will be recruited.

### Statistical Analysis

All statistical analyses will be performed using SPSS (version 20.0; IBM Corp). Multiple imputation will be used to address missing outcome data, with each missing value imputed 5 times and the mean of these imputations used for analysis. The intention-to-treat dataset will be used as the primary dataset. Feasibility outcomes will be primarily reported as observed proportions. The 95% CIs for feasibility outcomes will be calculated using Wilson score intervals and presented as a sensitivity analysis. Between-group differences in VAS scores, the WOMAC functional subscale, and credibility and expectancy scores will be assessed using either 2-tailed independent-sample *t* tests or Mann-Whitney *U* tests, as appropriate. Linear regression analyses will be conducted to examine the associations between within-group changes in VAS scores and skin temperature and their corresponding baseline values. Assessment of treatment effects will be exploratory. Results from the VAS and WOMAC will be used to estimate the effect size range for calculating the sample size of a definitive trial. Effect size will be quantified using Cohen *d*, with values of approximately 0.2, 0.5, and 0.8 representing small, medium, and large effects, respectively. All statistical tests will be 2-sided, with a significance level set at *P*=.05.

### Ethical Considerations

The study protocol was approved by the research ethics committee of the Third Affiliated Hospital of Beijing University of Chinese Medicine (BZYSY-2024KYKTPJ-44) and registered in the International Traditional Medicine Clinical Trial Registry (ITMCTR2025000019). Recruitment information will be disseminated through the acupuncture department and online media platforms. Participants will provide written informed consent. Original data will be stored using paper case report forms. Deidentified data will be used for storage and analysis.

## Results

**Figure 3:**
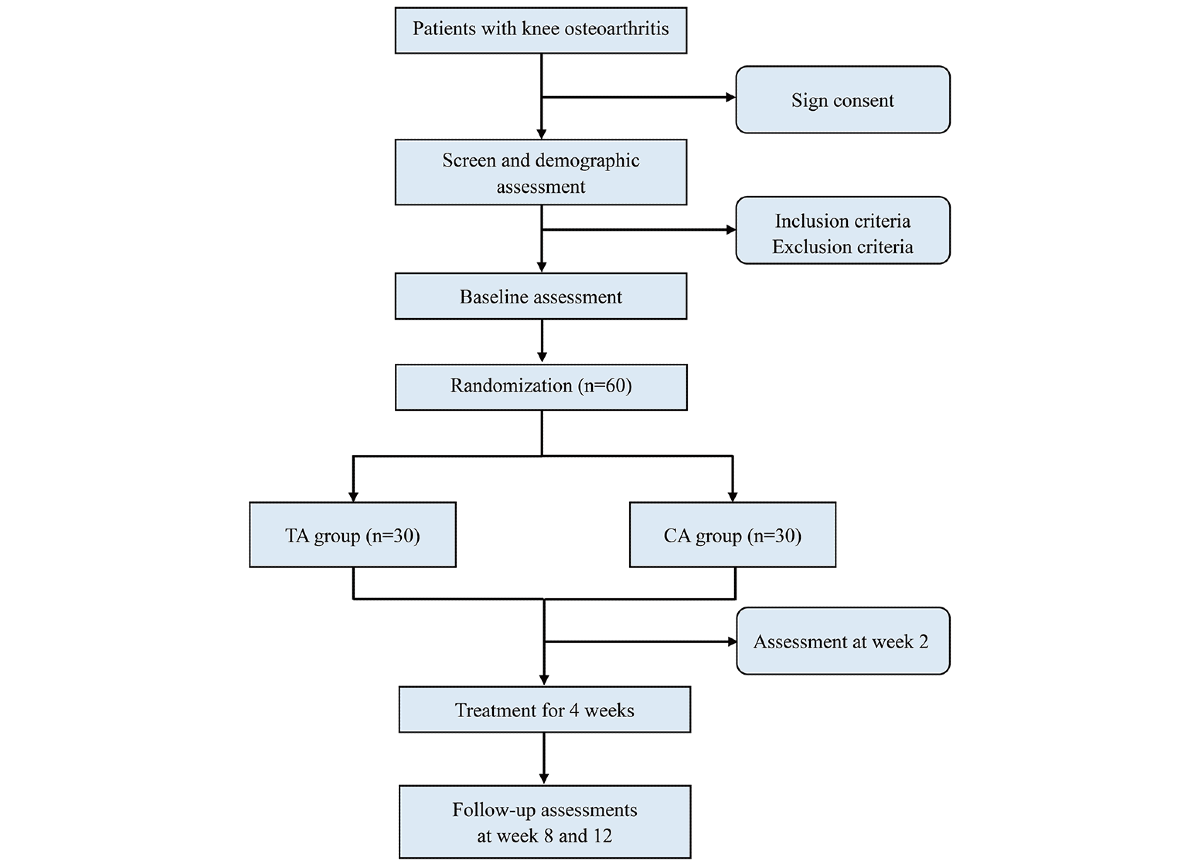
Flowchart of the study. CA: conventional acupoint; TA: thermal-sensitized acupoint.

Figure 3 shows the study flow diagram. The study was approved by the research ethics committee on November 15, 2024, and registered in the trial registry on January 6, 2025. Major funding was obtained on January 1, 2025. As the first participant was enrolled on February 25, 2025, a total of 30 participants had been enrolled by January 1, 2026. All enrollment and follow-up are expected to be completed by June 2026. Any protocol amendments will be documented and reported. The study results will be published in a peer-reviewed journal.

## Discussion

### Anticipated Findings

This study focuses on the treatment sites in acupuncture therapy, which were relatively underexplored in previous studies. A significant issue in acupuncture research is the substantial variability in acupoint selection, which often lacks objective justification. One reason for the limited acceptability of acupuncture is the reliance on acupoints derived from traditional theories, for which the biological rationale remains unclear [33]. Although the use of *ashi* points in traditional acupuncture or dry needling is supported by some biological rationale, these points are primarily chosen based on the patient’s subjective pain perception.

IRT can accurately identify areas of inflammation in the knee joint, and acupuncture guided by IRT offers a more objective approach to determining treatment sites. Compared with other acupuncture studies using TAs [34,35], a potential advantage of this study lies in individualized treatment guided by objective IRT measurements. Rather than directly adopting the TAs suggested in previous studies, treatment sites will be selected in a targeted manner according to individual differences. This acupuncture method, based on a simple imaging technique, could enhance the acceptability and dissemination of acupuncture in nontraditional Chinese medicine health care settings.

A limitation of this study is the lack of sufficient preliminary evidence to calculate the sample size, which means that the evaluation of efficacy differences between the two acupuncture therapies is a secondary aim. As a pilot trial, the study uses feasibility as the primary outcome. This primary outcome will be used to assess the multidimensional feasibility of conducting a large-scale study. Clinical effect and safety will be secondary outcomes, providing preliminary evidence. Another limitation is that, as a single-center study, patient characteristics may be limited.

### Conclusions

The findings of this study will provide feasibility data and preliminary evidence of clinical effect to support future clinical adoption of acupuncture prescription based on TAs for the treatment of KOA.

## Appendix

Multimedia Appendix 1 Standard Protocol Items: Recommendations for Interventional Trials (SPIRIT) guidelines checklist.

Multimedia Appendix 2 Information on acupoint selection methods.

## Abbreviations

CA: conventional acupoint
IRT: infrared thermography
KOA: knee osteoarthritis
SPIRIT: Standard Protocol Items: Recommendations for Interventional Trials
TA: thermal-sensitized acupoint
VAS: visual analog scale
WOMAC: Western Ontario and McMaster Universities Osteoarthritis Index

## Data Availability

Data sharing is not applicable to this article as no data sets were generated or analyzed during this study.
