# Supplementary material for: Acupuncture Prescription Based on Thermal-Sensitized Acupoints for the Treatment of Knee Osteoarthritis: Protocol for a Randomized Controlled Pilot Trial
Source: JMIR Res Protoc. 2026 Jan 28;15:e81837. doi: 10.2196/81837 (PMC12895158; doi:10.2196/81837)
Supplement: Multimedia Appendix 2 [file resprot_v15i1e81837_app2.docx]

**Supplementary Material 1**

**eTable 1.** Acupoints’ locations, corresponding needle type and depth of insertion

| **Acupoint** | **Name** | **Location** | **Needle type** | **Depth of insertion** |
| --- | --- | --- | --- | --- |
| Fixed acupoints | Dubi (ST35) | On the anterior aspect of the knee, in the depression lateral to the patellar ligament | 0.25×40mm | 25~40mm |
|  | Neixiyan (EX-LE5) | On the anterior aspect of the knee, in the depression medial to the patellar ligament | 0.25×40mm | 25~40mm |
|  | Ququan (LR8) | On the medial aspect of the knee, in the depression medial to the tendons of the semitendinosus and the semimembranosus muscles, at the medial end of the popliteal crease | 0.25×25mm | 20~25mm |
|  | Xiyangguan (GB33) | On the lateral aspect of the knee, in the depression between the biceps femoris tendon and the iliotibial band, posterior and proximal to the lateral epicondyle of the femur | 0.25×40mm | 25~40mm |
|  | Ashi point | The point where the patient feels most pain | depends on the location | |
| Optional acupoints for yangming meridian syndrome | Futu (ST32) | On the anterolateral aspect of the thigh, on the line connecting the lateral end of the base of the patella with the anterior superior iliac spine, 6 cun^a^ superior to the base of the patella | 0.25×40mm | 25~40mm |
|  | Liangqiu (ST34) | On the anterolateral aspect of the thigh, between the vastus lateralis muscle and the lateral border of the rectus femoris tendon, 2 cun superior to the base of the patella | 0.25×40mm | 25~40mm |
|  | Heding (EX-LE2) | On the anterior aspect of the thigh, in the depression superior to the base of the patella | 0.25×25mm | 13~20mm |
|  | Zusanli (ST36) | 3 cun directly below ST35, and one finger-breadth lateral to the anterior border of the tibia | 0.25×50mm | 25~50mm |
|  | Fenglong (ST40) | On the anterolateral aspect of the leg, lateral border of the tibialis anterior muscle, 8 cun superior to the prominence of the lateral malleolus | 0.25×40mm | 25~40mm |
| Optional acupoints for three-yin meridian syndrome | Xuehai (SP10) | On the anteromedial aspect of the thigh, on the bulge of the vastus medialis muscle, 2 cun superior to the medial end of the base of the patella | 0.25×40mm | 25~40mm |
|  | Yingu (KI10) | On the posteromedial aspect of the knee, just lateral to the semitendinosus tendon, in the popliteal crease | 0.25×40mm | 25~40mm |
|  | Yinlingquan (SP9) | On the tibial aspect of the leg, in the depression between the inferior border of the medial condyle of the tibia and the medial border of the tibia | 0.25×50mm | 25~50mm |
|  | Xiguan (LR7) | On the tibial aspect of the leg, inferior to the medial condyle of the tibia, 1 cun posterior to SP9 | 0.25×40mm | 25~40mm |
|  | Sanyinjiao (SP6) | On the tibial aspect of the leg, posterior to the medial border of the tibia, 3 cun superior to the prominence of the medial malleolus | 0.25×40mm | 25~40mm |
|  | Taixi (KI3) | On the posteromedial aspect of the ankle, in the depression between the prominence of the medial malleolus and the calcaneal tendon | 0.25×25mm | 13~25mm |
|  | Taichong (LR3) | In the depression anterior to the junction of the first and second metatarsal bones | 0.25×25mm | 13~25mm |
|  | Gongsun (SP4) | On the medial aspect of the foot, anteroinferior to the base of the first metatarsal bone, at the border between the red and white flesh | 0.25×25mm | 13~25mm |
| Optional acupoints for taiyang meridian syndrome | Weiyang (BL39) | On the posterolateral aspect of the knee, just medial to the biceps femoris tendon in the popliteal crease | 0.25×40mm | 25~40mm |
|  | Weizhong (BL40) | On the posterior aspect of the knee, at the midpoint of the popliteal crease | 0.25×40mm | 25~40mm |
|  | Chengshan (BL57) | On the posterior aspect of the leg, at the connecting point of the calcaneal tendon with the two muscle bellies of the gastrocnemius muscle | 0.25×50mm | 25~50mm |
|  | Kunlun (BL60) | On the posterolateral aspect of the ankle, in the depression between the prominence of the lateral malleolus and the calcaneal tendon | 0.25×25mm | 13~20mm |
| Optional acupoints for shaoyang meridian syndrome | Fengshi (GB31) | On the lateral aspect of the thigh, in the depression posterior to the iliotibial band where the tip of the middle finger rests, when standing up with the arms hanging alongside the thigh | 0.25×50mm | 25~50mm |
|  | Yanglingquan (GB34) | On the fibular aspect of the leg, in the depression anterior and distal to the head of the fibula | 0.25×40mm | 25~40mm |
|  | Waiqiu (GB36) | On the fibular aspect of the leg, anterior to the fibula, 7 cun proximal to the prominence of the lateral malleolus | 0.25×40mm | 25~40mm |
|  | Xuanzhong (GB39) | On the fibular aspect of the leg, anterior to the fibula, 3 cun proximal to the prominence of the lateral malleolus | 0.25×25mm | 13~20mm |
|  | Zulinqi (GB41) | On the dorsum of the foot, distal to the junction of the bases of the fourth and fifth metatarsal bones, in the depression lateral to the fifth extensor digitorum longus tendon | 0.25×25mm | 8~13mm |

^a^ 1 cun (≈20 mm) is defined as the width of the interphalangeal joint of patient’s thumb.

**IRT data acquisition and extraction specification**

**1 Data acquisition specification**

1. Record personal information of all participants in data sheets.

2. Consummation of alcohol within 4 hours before the measurement is not allowed for participants.

3. Conduction of vigorous exercise, or to events that may severely interfere with skin temperature, such as electrotherapy, ultrasound, heat or cold exposure within two hours prior to measurement are not allowed.

4. Control the ambient temperature at 25±2 ℃, and control the relative humidity of the air at 40%-50%.

5. In the field angle of the infrared thermal image, avoid interference heat source and obvious air flow.

6. The IRT detection equipment is a portable medical infrared thermal imager. The thermal sensitivity is 0.05℃ at 30℃, the spectral range is 7.5-13 μm, the image pixel size is 256×336, and the acquisition frequency is 25 Hz.

7. To make the equipment work in a steady state, power the infrared thermal imager 0.5 hours before data collection.

8. Set the vertical distance between the participants and the camera at 1.5 meters.


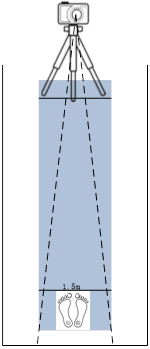


9. Perpendicular the centerline of the lens field angle to the human body by a liftable gimbal.

10. Set the emissivity of the detector at 0.98 (default value).

11. Collect the image at 9:00-16:00 for all participants.

12. Make sure all participants stay in the same posture.

13. The data file is filled in a 16-bit integer data format and stored in *.dat.

14. Separate the image collecting site from the outside environment by a movable screen.

15. When removing the lower clothes, keep participants’ underwear to maximize their privacy.

**2 Data extraction specification**

1. Draw ROIs on the IRT image on the side with more severe symptoms.

2. The size of the ROI is a circular area with a radius of 3 pixels centered on the acupoint positioning (fixed by software).

3. Extract the average value of the temperature from the ROI.

4. Draw the ROI by 2 researchers independently. If the skin temperature of the acupoint is inconsistent, ask for a third researcher for discussing the ROI location.
